# Assessment of Different Infectious Bovine Rhinotracheitis Marker Vaccines in Calves

**DOI:** 10.3390/vaccines10081204

**Published:** 2022-07-28

**Authors:** Stefano Petrini, Alessandra Martucciello, Cecilia Righi, Giovanna Cappelli, Claudia Torresi, Carlo Grassi, Eleonora Scoccia, Giulia Costantino, Cristina Casciari, Roberto Sabato, Monica Giammarioli, Esterina De Carlo, Francesco Feliziani

**Affiliations:** 1 National Reference Centre for Infectious Bovine Rhinotracheitis (IBR), Istituto Zooprofilattico Sperimentale Umbria-Marche, “Togo Rosati,” 06126 Perugia, Italy; c.righi@izsum.it (C.R.); c.torresi@izsum.it (C.T.); e.scoccia@izsum.it (E.S.); g.costantino@izsum.it (G.C.); c.casciari@izsum.it (C.C.); r.sabato@izsum.it (R.S.); m.giammarioli@izsum.it (M.G.); f.feliziani@izsum.it (F.F.); 2 National Reference Centre for Hygiene and Technology of Breeding and Buffalo Production, Istituto Zooprofilattico Sperimentale del Mezzogiorno, 84131 Salerno, Italy; alessandra.martucciello@izsmportici.it (A.M.); giovanna.cappelli@izsmportici.it (G.C.); grassicarlo@libero.it (C.G.); esterina.decarlo@izsmportici.it (E.D.C.)

**Keywords:** calves, IBR, marker vaccines, latency

## Abstract

Three commercially available infectious bovine rhinotracheitis (IBR) live marker vaccines were evaluated for their ability to provide clinical protection to vaccinated calves against wild-type (wt) Bovine alphaherpesvirus-1 (BoHV-1) challenge and their possible effect on wt BoHV-1 latency reactivation following the challenge. On 35 post-vaccination days (PVDs), all animals were challenged with wt BoHV-1. Only the calves in the control group developed severe forms of IBR. The reactivation of latent BoHV-1 was induced by dexamethasone (DMS) treatment on 28 post-challenge days (PCDs). All animals showed IBR clinical signs on three post-DMS treatment days (PDTDs). On PVD 14, all vaccinated animals developed neutralizing antibodies (NAs), whereas in control animals, the NAs appeared post-challenge. The positivity for glycoprotein-B (gB) was detected using real-time polymerase chain reactions in all animals from PCDs 1 to 7. In contrast, the gB-positivity was observed in the immunized calves from PDTDs 3 to 10. Positive expression of gD and gE was observed in nasal swabs of all calves on PDTD 7. These findings suggested that the IBR marker vaccines evaluated in this study protected against wt BoHV-1-induced disease but not against wt BoHV-1-induced latency reactivation, indicating the necessity of developing new products to protect animals from wt BoHV-1-induced latency.

## 1. Introduction

Bovine alphaherpesvirus-1 (BoHV-1) is one of the main causative agents of different respiratory (infectious bovine rhinotracheitis, IBR), genital diseases (infectious pustular vulvovaginitis, IPV; infectious balanoposthitis, IBP), and other clinical conditions, such as conjunctivitis, encephalitis, endometritis, infertility, abortions, and enteritis [1].

After an acute phase of infection, the virus localizes to the sensory nervous ganglia (trigeminal or sacral) or tonsils, establishing a state of viral latency [2]. The epizootiological significance of latency is the persistence of the virus in the cattle population by silent carriers without evidence of clinical disease. In these animals, 

Following various immunosuppressive stimuli, such as viral superinfection, *Dictyocaulus viviparus* infestation, injection of glucocorticoids, and partum, the latent virus can be reactivated, re-excreted, and cause serious clinical respiratory (IBR) or genital (IPV, IBP) outbreaks, leading to severe economic losses [3,4,5]. 

To reduce the viral circulation of BoHV-1 infection and, consequently, the occurrence of outbreaks in the European Union (EU), conventional or new-generation marker vaccines (aka DIVA vaccines that differentiate vaccinated from infected animals) are used [6,7,8]. 

Compared with traditional products, marker vaccines offer the advantage of distinguishing between vaccinated (gE-negative) and infected animals (gE-positive) [9]. In particular, two types of marker vaccines [10] are commercially available in Europe: (i) a single deletion of the gene encoding the glycoprotein E (gE) of BoHV-1, and (ii) a double deletion of the genes encoding both gE and the thymidine-kinase enzyme (tk). 

The choice of vaccine type is the key to the success of an IBR eradication program that involves immunization. In particular, the vaccine must protect animals from infection and the development of clinical signs of the disease while minimizing wild-type (wt) BoHV-1 latency as much as possible.

Several IBR marker vaccines have been developed, and their efficacy in providing clinical protection has generally been studied in independent, unconnected experiments. However, for some IBR marker vaccines, it is difficult to draw a consistent conclusion on their ability to provide clinical protection [10]. Moreover, the knowledge of the ability of these marker vaccines to protect against wt BoHV-1-induced latency reactivation is limited [11].

Therefore, we hypothesized that experimental evaluation of different IBR marker vaccines following a challenge with wt BoHV-1 could unravel the potential of the vaccines to protect calves. Furthermore, an assessment of the possible effects of these marker vaccines on wt BoHV-1 latency reactivation following such a challenge could be helpful in the strategical development of IBR eradication programs. To test these hypotheses, the present study aimed to compare the ability of three live marker vaccines to clinically protect calves against experimental infection with wt BoHV-1 and evaluate their potential to protect animals against wt BoHV-1-induced latency.

The findings of this study could help in designing safe and effective IBR eradication strategies, especially in the context of protection against wt BoHV-1-induced latency.

## 2. Materials and Methods

### 2.1. Virus

The wt strain 16453/07 TN of BoHV-1 isolated during an IBR outbreak in a dairy herd in central Italy in 2007 (Petrini, unpublished data) was used in this study. The strain was used at the fourth passage on Madin–Darby Bovine Kidney (MDBK) cell cultures at a titer of 10^7.00^ median tissue culture infectious dose (TCID_50_/mL).

### 2.2. Vaccines

Three marker vaccines were used in this study (Table 1). Two doses (2 mL each) of these vaccines were administered to each animal 28 days apart. The calves received the first dose of the vaccine at the age of 3 months. Three calves that did not receive any vaccine were used as the controls.

### 2.3. Experimental Design

Twelve Friesian calves devoid of BoHV-1 neutralizing antibodies (NAs) were used in this study. All animals were selected from a dairy herd in Southern Italy (Campania region). According to farm records, no vaccine against BoHV-1 had been used before, and no recent history of respiratory disease was registered. The selected animals were transferred to an experimental farm in the Istituto Zooprofilattico Sperimentale Mezzogiorno (Salerno Province). The calves were fed twice daily with hay, concentrated fattening feed, and water ad libitum. The maintenance and experimental protocols were according to European legislation on the protection of animals used for scientific purposes [12]. Furthermore, the Italian Ministry of Health approved the experiments under authorization number 835/2019-PR. 

The number of animals in each group was determined through the sampling procedure envisaged in an experimental clinical study to compare proportions in terms of superiority, with an error of 5% and a study power of 80%. For the proportion of the appearance of the event, 0% and 90% were considered in the control and experimental groups, respectively. The calves were divided into four groups of three animals each and used as described in the following sections [13].

#### 2.3.1. Vaccination and Challenge Infection

The first three groups were immunized with vaccines A, B, and C, respectively (Table 1), and the fourth group served as an unvaccinated control group. The immunized animals were observed for 30 days following vaccination, during which their temperatures were recorded daily for 15 days, and each calf was examined for the appearance of clinical signs. Any possible adverse reactions after vaccination were monitored by a veterinary practitioner. On the day of the first vaccination (time 0) and 14, 21, and 28 post-vaccination days (PVDs), serum samples were collected from each calf and tested for BoHV-1 antibodies. Nasal swabs in transport fluid composed of Minimum Essential Medium (MEM) were obtained from each calf on 0, 1, 2, 3, 4, 6, 7, 10, and 14 PVDs, and used for virological investigations using real-time polymerase chain reaction (PCR). 

Thirty-five days after the first immunization, all animals were subjected to challenge infection with a wt BoHV-1 strain. Each calf received 5 mL × 10 ^7.00^ TCID_50_/mL administered via the intranasal route. 

The calves were kept under observation for vaccination and experimental infection stages, and the rectal temperatures were recorded daily. Serum samples were collected from all animals on 0, 7, 14, and 21 post-challenge days (PCDs) and tested for BoHV-1 antibodies. Nasal swabs in transport fluid MEM were obtained from each calf on 1, 2, 3, 4, 7, and 14 PCDs and used for virological investigation using real-time PCR. 

#### 2.3.2. Wild-Type BoHV-1 Reactivation

Twenty-eight PCDs, the calves were subjected to dexamethasone (DMS, Dexadreson^®^, MSD Animal Health S.r.l., Milan, Italy) treatment. Each calf received 0.1 mg DMS/kg body weight by the intravenous route for five consecutive days and was observed for the next 20 days for clinical responses. The nasal swabs for virological investigations (real-time PCR) were collected on 0, 1, 2, 3, 4, 7, 10 and 14 PDTDs. Serum samples for serological testing were collected on 0 and 14 PDTDs. 

### 2.4. Blood Samples Collection

Blood samples (approximately 8 mL from each calf) were collected from the coccygeal or jugular veins. All samples were transported to the laboratory under refrigeration within 2 h of collection before testing and stored at −20 °C until further processing. Afterward, the blood samples were centrifuged at 850× *g* for 30 min at 4 °C to extract the serum for serological investigations.

### 2.5. Neutralization Test

Serum samples were tested using a protocol described earlier [14]. Briefly, 50 µL of undiluted serum samples and two-fold dilutions of each serum sample were mixed with 50 µL of 100 TCID_50_ of BoHV-1 (Los Angeles reference strain 01/17) and transferred into three wells of 96-well microtiter plates (CytoOne ^®^, Starlab, Milan, Italy). The 96-well plates with the mixture were incubated at 37 °C for 24 h. Then, 30,000 MDBK cell cultures in 100 µL MEM were added to each well and incubated for 4 days at 37 °C. The cells were obtained from Biobanking of Veterinary Resources (BVR; Brescia, Italy) and identified with the code BS CL 63. After the incubation, the plates were examined using an inverted tissue culture microscope (Olympus IX51, Olympus Corporation, Tokyo, Japan) to determine the cytopathic effect. Neutralization titers were expressed as the highest dilution that inhibited cytopathology.

### 2.6. ELISA Tests

Two commercial ELISA tests (IDEXX IBR gB X3 Ab, Westbrook, ME, USA; and IDEXX IBR gE Ab test, Westbrook, ME, USA) were used to examine the collected sera following the protocols supplied with the kits. The results are expressed according to the manufacturer’s instructions. The microplates were read using an automated plate reader (Sunrise^TM^, Tecan AG, Männedorf, Switzerland), and the data were analyzed using the Magellan software (Tecan AG, Männedorf, Switzerland).

### 2.7. Real-Time PCR

Viral DNA was extracted from nasal swabs using a High Pure PCR Template Preparation Kit (Roche Diagnostics Deutschland, Mannheim, Germany) following the manufacturer’s instructions. To investigate the presence of BoHV-1, gB-specific real-time PCR was performed as described elsewhere [14] with a modified endogenous control amplification. In particular, herpes simplex gB-specific amplification was merged with genomic β-actin amplification using primers and probes described by Wernike et al. [15]. The probe was modified as follows: 

5′-VIC-TCG CTG TCC ACC TTC CAG CAG ATG T-TAMRA-3

All samples were tested in duplicate and considered positive when the Ct value was equal to or less than 45. To discriminate between the presence of wt BoHV-1 (gE-positive) or marker vaccine virus (gE-negative), we performed gD- and gE-specific triplex real-time PCR as previously described [15]. All samples were tested in duplicate and considered positive when the Ct value was equal to or less than 40.

### 2.8. Statistical Analysis

The titers of NA responses were measured as log_10_. The average value titers were calculated for each animal group and all sampling times. The Wilcoxon Mann–Whitney non-parametric test was used to evaluate the statistically meaningful differences in rectal temperature, clinical signs, and NAs in each vaccinated group after evaluating the normality of the data with the Shapiro–Wilk test. Differences were considered significant at *p* ≤ 0.05. The analysis was performed using Stata v.11.2 software (StataCorp LCC, College Station, TX, USA).

## 3. Results 

### 3.1. Clinical Response

The tested vaccines did not cause any adverse reactions in the immunized calves within the 35 PVDs. Only two calves in Group A and one calf in Group B developed a cough and were treated with antibiotics (procaine benzylpenicillin combined with dihydrostreptomycin) for four days. Rectal temperatures in the vaccinated groups were within the normal values and similar to those in the control group. 

After challenge infection, no clinical signs were observed in any of the immunized calves. In contrast, in the unvaccinated controls, on PCD 4, two animals showed nasal mucopurulent exudate, dyspnea, and cough. In addition, lesions in the nasal mucosa, consisting of pseudomembranes, were observed for at least 4 to 8 days. In addition, in unvaccinated controls, rectal temperatures increased up to 41.0–41.5 °C from PCD 2 to PCD 8.

All calves showed clinical signs (pyrexia, anorexia, dyspnea, increased respiratory rate, and cough) on PDTD 3. The calves in Group A showed bilateral nasal discharge that lasted 1–3 days. In addition, two calves in Group B, and all calves in Group C and the control group showed bilateral nasal discharge. In these calves, lesions in the nasal mucosa consisting of pseudomembranes, pustules, tracheitis, and typical breath snores were observed for at least 3–5 days.

Two calves, including one each in the control group and Group B, died during the vaccination period. 

Necropsy of these calves revealed that the sudden death of the calf in the control group (on PVD 3) was due to cardiac tamponade, whereas that of the other calf belonging to Group B (on PVD 30) was due to acute pericarditis.

### 3.2. Serological Investigations 

The BoHV-1 NA titer was detected in vaccinated animals on PVD 14, presenting a mean titer of 1.30 log_10_ (Group A, *p* = 0.20) or 1.20 log_10_ (Group B, *p* = 0.20) and 0.70 log_10_ (Group C; *p* = 0.20). The NA titer of the vaccinated animals progressively increased up to PVD 28, presenting a mean titer of 2.71 log_10_ (Group A, *p* = 0.20) or 1.51 log_10_ (Group B, *p* = 0.20) and 2.00 log_10_ (Group C; *p* = 0.20). On PVD 14, the vaccinated calves were seropositive for gB-ELISA. All calves tested negative for gE-ELISA during the vaccination period. Seroconversion was not detected in the unvaccinated controls (Table 2).

The NA titer of the vaccinated animals increased after PCD 14, with a mean titer of 3.51 log_10_ (Group A, *p* = 0.40) or 3.46 log_10_ (Group B, *p* = 0.66) and 3.41 log_10_ (Group C; *p* = 0.60). Only the animals in Group A showed a decrease in titer by 0.10 log_10_ on PCD 21. In the unvaccinated controls, NAs appeared for the first time on PCD 7, with a mean titer of 1.35 log_10_. This value increased to 3.16 log_10_ on PCD 21. After challenge infection, the vaccinated calves remained positive for gB-ELISA, whereas those in the control group seroconverted on PCD 14. All animals were seroconverted to gE-ELISA on PCD 14 (Table 2).

On the day of the start of DMS treatment, all calves had antibodies to BoHV-1 3.41 log_10_ (Group A, *p* = 0.60), 3.31 log_10_ (Group B, *p* = 1.00), and 3.21 log_10_ (Group C; *p* = 1.00). In Groups A and C, the titer was decreased by 0.30 log_10_ and 0.10 log_10_, respectively, on PDTD 14. The titer remained the same in the unvaccinated controls (3.16 log_10_) during the entire DMS treatment period (Table 2).

### 3.3. Virological Investigations 

During the vaccination period, no positivity to gB real-time PCR was detected in any animal. Unlike during the challenge infection, positivity to gB real-time PCR from PCD 1 to PCD 7 was detected in all animals. In general, during this last phase, in the immunized animals, the Ct values ranged from 22.82 to 37.13, whereas in the unvaccinated controls, those varied from 20.56 to 30.28 (Table 3).

Following the DMS treatment, a positive signal to gB real-time PCR was observed in all vaccinated calves, except for one calf (# 563) in Group A that tested negative. In Group A, a positive real-time PCR was observed from PDTD 3 to PDTD 7, whereas in calves of Groups B and C, a positive real-time PCR was detected from PDTDs 4 to 10 and PDTDs 4 to 7, respectively. In the control group, the animals were positive for gB real-time PCR from PCD 2 to PCD 10, and a positive real-time PCR was observed from PDTD 2 to PDTD 10. After DMS treatment, the Ct values in vaccinated calves ranged from 22.84 to 39.07, while in the unvaccinated calves, they ranged from 22.13 to 38.79 (Table 3). 

As shown in Table 4, following PDTD 7, a positive result for gD and gE according to real-time PCR was observed in all calves, except for one calf (# 563) in Group A. The Ct values ranged from 22.27 to 39.65 in the immunized calves, while those in the unvaccinated calves varied from 21.81 to 31.71 (Table 4). 

## 4. Discussion

The European Legislation (EU) 429/2016, the so-called “Animal Health Law” [16], entered into force in April 2021, and subsequent regulations [17,18] have been issued, providing the European Member States with the opportunity to submit control or eradication plans for different infectious diseases, including IBR. For control or eradication plans for IBR infection with a high prevalence, the DIVA strategy is applied [6,19] using IBR marker vaccines. Furthermore, the European group studying IBR—as part of the “DISease CONtrol TOOLS” (DISCONTOOLS)—suggests that live IBR marker vaccines may not protect animals from latency induced by wt BoHV-1 [20]. This indicated the emergency need to identify products that can protect the immunized calves against wt BoHV-1 latency reactivation, given the economic burden of latency reactivation. Therefore, in this study, we tested three different commercially available marker vaccines (single or double deletions). We hypothesized that they may induce clinical protection in calves after the wt BoHV-1 challenge and protect them against wt BoHV-1 latency reactivation following such a challenge. 

Regarding the first hypothesis, our findings showed that the vaccines used in these experiments were safe and effective in preventing clinical signs of disease following the wt BoHV-1 challenge. After challenge infection, the rectal temperatures of the vaccinated animals were normal and did not show any clinical symptoms compared with those of the control animals. In contrast, the unvaccinated control animals showed a typical clinical form of IBR on PCD 4. Furthermore, like the control animals, all immunized animals showed viral shedding from PCDs 1 to 7. These results were similar to those reported in previous studies on the safety and efficacy of traditional and marker vaccines (single or double deletions) for cattle and buffaloes [21,22,23,24,25,26]. On the contrary, a study by Kaashoek et al. demonstrated the post-challenge development of small lesions of the nasal mucosae in calves immunized with gE-negative and gE/tk-negative vaccines [26]. These small lesions were observed less often in gE/tk-negative vaccine-infected calves compared to those in gE-negative infected calves [27]. Furthermore, clinical protection was not complete in animals immunized with an inactivated product or BoHV-1 subunit vaccines, and virus shedding by vaccinated calves continued for several days after challenge infection [28]. However, the results obtained in this study were supported by serological data obtained on the day of the challenge. All the vaccines tested induced a high level of NAs (Group A = 2.71 log_10_; Group B = 1.51 log_10_; Group C = 2.00 log_10_) to protect calves from clinical signs of disease after challenge with the wild-type strain of BoHV-1 (16453/07 TN). In contrast, in a study conducted by Mahan et al., only 8 out of 18 vaccinated calves showed NAs after intranasal administration of a modified live vaccine that included a *ts*-strain of BoHV-1. The NA titers against BoHV-1 were 1:8 or lower [29]. In addition, a positive result was observed in calves vaccinated with gB-ELISA on PVD 14. In contrast, in the control group, gB-positivity was observed on PCD 14. These results were similar to those published for cattle and buffalos in other studies, and demonstrated that these marker vaccines administered to calves or buffalos constantly increased their humoral immune response after the challenge. The controls seroconverted to gB-ELISA and NA after PCD 14 [8,21,22]. The results obtained using gE-ELISA showed that all animals were seroconverted on PCD 14. These results agreed with those obtained previously, which showed that seroconversion to the gE protein occurred 2–4 weeks after infection [8,10,30]. Moreover, the gE antibodies were seroconverted in all groups from PCDs 7 to 14. The gE forms a heterodimer with gI and constitutes a fragment crystallizable (Fc) receptor involved in the disruption of the host immune response. Furthermore, the gE–gI complex of BoHV-1 or bovine alphaherpesvirus 5 (BoHV-5) [31,32] facilitates the basolateral spread of progeny viruses in polarized cells, suggesting its role in virion cell-to-cell spread and neurovirulence [33,34,35,36]. Studies have also shown that Suid alphaherpesvirus (SuHV-1) or herpes simplex virus (HSV), gE, or gI minor mutants had significantly reduced neurovirulence, and their ability to infect neurons after nasopharyngeal or ocular infection was significantly reduced [37,38,39]. Taken together, the experimental evaluation of the marker vaccines supported the first hypothesis. It demonstrated that the tested vaccines protected immunized calves against the clinical signs of the disease induced by the wt BoHV-1 challenge. 

Concerning the second hypothesis, we showed that after DMS treatment, all calves immunized with IBR marker vaccines (single or double deletions) reactivated the wild-type BoHV-1 virus used for challenge infection. DMS treatment has two effects on the reactivation of wt BoHV-1 latency: (1) direct stimulation of viral gene expression and replication, which interfere with key antiviral innate immune responses; and (2) immune suppression enhances viral spread, transmission, and clinical signs of the disease [40,41,42]. However, to date, the pathogenic mechanisms involved in the protection of the marker vaccine against reactivation of wt BoHV-1 after DMS treatment are not fully understood. In an uninfected organism, wt BoHV-1 in the upper respiratory tract (mucous membranes) directly penetrates the peripheral nerve endings present on the respiratory mucosa and retrogrades along the microtubes of the axon using cutaneous receptors, moves into the trigeminal nervous ganglia, and induces latency [42]. The virus synthesizes a few latency-associated transcript (LAT) loci during this phase, including the latency-related (*LR*) gene and its open reading frames (ORFs) [42,43]. Various immunosuppressive stimuli, such as DMS treatment, food and water deprivation during the shipping of cattle, weaning, and dramatic weather changes, facilitate the binding of corticosteroids to receptors for glucocorticoids and mineralocorticoids, which reactivates latent BoHV-1 and further activates the expression of different viral genes such as *bICP*40 and a few late genes during productive infection [42]. Reportedly, when the virus was reactivated, in the first-hour post-DMS treatment, *bICP4* RNA and protein were expressed in vitro, but no other viral genes such as *bICP0* or *bICP22* were stimulated, while at four hours post-treatment, late genes such as *UL10*, *UL16*, and *UL17* were activated [43]. It has been shown that DMS treatment stimulates the expression of protein kinase serum glucocorticoid receptor kinase 1 (SKG1), which is responsible for productive infection [42,43]. During the reactivation phase from latency, anterograde transport of the virus through nerve fibers to the mucosal membrane might be impaired, and a second immune response releases interferons and activates T and B cells [44,45]. 

The wt BoHV-1 virus was detected using real-time PCR for gD and gE from all vaccinated calves on PDTDs 3–10, while in the control calves, the same positive result was observed on PDTD 2. Furthermore, this positivity was associated with severe clinical symptoms of respiratory diseases. In Group A, wt BoHV-1 was reactivated from PDTDs 3 to 7. However, only one calf in Group A did not shed any virus during DMS treatment. This result suggested that the vaccine administered to the first group (double deletion) in a limited number of immunized animals at least partially protected the calves against wt BoHV-1-induced latency. However, these results should be investigated in further experiments involving a large group of animals. Likewise, the calves in Group B discharged wt BoHV-1 from PDTDs 4 to 10, while the animals in Group C reactivated the virus from PDTDs 4 to 7. In contrast, the animals in the control group excreted the virus from PDTDs 2 to 10. These results were concordant with those obtained in other studies that reported the excretion of the virus from PDTDs 5 to 10 [21,22,28,41]. Collectively, these results demonstrated that the tested vaccines did not protect immunized calves from wt BoHV-1 challenge-induced latency. 

The present study had some limitations. The cytokines and chemokines activated during the challenge, infection, and viral reactivation phases were not investigated. It is known that an increase in the expression of chemokines and CD4^+^, CD8^+^, and T cells is observed when the virus infects the ganglion, which is responsible for controlling viral replication [42,43]. Furthermore, only a small number of animals could be used due to the current European legislation on animal testing, which requires a reduced number of animals based on the 3 Rs principle [12]. Nevertheless, the number of calves selected in this study was sufficient to obtain statistically significant results.

The recombination is a mechanism of genetic variation in herpesviruses [46]. This phenomenon is represented by homologous recombination (intramolecular) and occurs during co-infection or delayed infection within a short time interval. In nature, this type of recombination is little known. However, further studies have shown that recombination occurs when two different viral strains of the same herpesvirus species are co-inoculated [47,48]. In addition, recombinant herpesviruses have been detected both during primary infection and after reactivation from latency [49]. Several studies demonstrated the virulence of a gE-negative recombination and genetic recombination between gE-negative recombinant viruses and wt BoHV-1 in each co-infection situation [50,51]. Although we did not evaluate the genomic composition of the re-isolated viruses, the findings of the present study, together with those of the studies described above, indicated that the viruses may have modified or recombined their viral genome. However, further studies employing next-generation sequencing technologies to sequence the viruses identified after DMS treatment are required to confirm these speculations. 

Furthermore, one of the methods to reduce the risk of infection, reactivation, and recombination of herpesviruses is the administration of vaccines via the intranasal (IN) route. It is well known that the immunity produced after the injection of IN vaccines can protect calves for several months. Moreover, this immunization route can evoke interferon production within 40 h after administration, thus providing antiviral action and stimulating the immune system. In addition, unlike intramuscularly administered vaccines, IN products do not induce immunosuppression [10]. Therefore, the potential of IN vaccines against wt BoHV-1 latency reactivation should be evaluated. 

## 5. Conclusions

The findings of the present study demonstrated that the marker vaccines evaluated here were safe and effective for the prevention of wt BoHV-1-induced clinical disease. However, they were not efficient in protecting the calves against latency reactivation induced by wt BoHV-1. These findings indicated the urgency of further studies to develop and evaluate new products to protect animals from wt BoHV-1-induced latency. Future research will also consider the study of important cytokines that either directly or indirectly inhibit virus replication by activation of effector cells (T-cell memory), as the cell-mediated response is a critical defense mechanism, and cell-to-cell spread occurs before hematogenous spread. Moreover, the induction of a robust T-cell memory is crucial for the long-term durability of immunity.

## Figures and Tables

**Table 1:** Bovine alphaherpesvirus-1 (BoHV-1) vaccines used in the experiment.

No. of Calves	Vaccine Identification	Type	Virus Concentration in TCID_50_ in One Dose (2 mL)	Inoculation Route
3	A	gE, tk, negative, modified live	10^7.74^	Intramuscular
3	B	gE negative, modified live	10^8.24^	Intranasal
3	C	gE negative, modified live	10^8.50^	Intramuscular
3	None	*Unvaccinated controls*		

gE, glycoprotein E of Bovine alphaherpesvirus-1; tk, thymidine-kinase enzyme of Bovine alphaherpesvirus-1; TCID_50_/mL, median Tissue Culture Infectious Dose.

**Table 2:** Antibody response of calves immunized with different BoHV-1 marker vaccines, challenge infected with virulent strain of BoHV-1, and treated with dexamethasone (DMS).

Group *		Post-Vaccination Day (PVD)				Post-Challenge Day (PCD)				Post-DMS Treatment Day (PDTD)	
		0	14	21	28	0 **	7	14	21	0 ***	14
A	gE-ELISA ^a^	-	-	-	-	-	-	+	+	+	+
	gB-ELISA ^b^	-	+	+	+	+	+	+	+	+	+
	NA ^c,d^	<1	1.30	1.30	2.71	2.71	3.51	3.51	3.41	3.41	3.11
	*p*-value	-	0.20	0.20	0.20	0.20	0.20	0.40	0.60	0.60	1
B	gE-ELISA ^a^	-	-	-	-	-	-	+	+	+	+
	gB-ELISA ^b^	-	+	+	+	+	+	+	+	+	+
	NA ^c,d^	<1	1.20	1.40	1.51	1.51	3.16	3.46	3.46	3.31	3.31
	*p*-value	-	0.20	0.20	0.33	0.33	0.33	0.66	1	1	1
C	gE-ELISA ^a^	-	-	-	-	-	-	+	+	+	+
	gB-ELISA ^b^	-	+	+	+	+	+	+	+	+	+
	NA ^c,d^	<1	0.70	0.96	2.01	2.01	3.21	3.41	3.41	3.21	3.11
	*p*-value	-	0.20	0.20	0.20	0.20	0.20	0.60	0.60	1	1
*Unvaccinated* *Controls*	gE-ELISA ^a^	-	-	-	-	-	-	+	+	+	+
	gB-ELISA ^b^	-	-	-	-	-	-	+	+	+	+
	NA ^c,d^	<1	<1	<1	<1	<1	1.35	2.86	3.16	3.16	3.16

* See Table 1 for vaccine identification; ** PCD 0 corresponds to PVD 35; *** PDTD 0 corresponds to PCD 28; ^a^ IDEXX IBR gE Ab test, Maine, USA; ^b^ IDEXX IBR gB X3 Ab, Maine, USA; ^c^ NA, neutralizing antibody; ^d^ expressed as log_10_ of the reciprocal of the highest dilution inhibiting cytopathology effect (mean value); *p*-value indicates the significant differences in NA titer between vaccinated calves and unvaccinated controls.

**Table 3:** Results of BoHV-1 detection by gB real-time PCR of calves immunized with different BoHV-1 marker vaccines, challenge infected with virulent strain of BoHV-1, and treated with DMS.

Group *	Identification No. of Calves	Post-Challenge Day (PCD)							Post-DMS Treatment Day (PDTD)							
		0 **	1	2	3	4	7	14	0 ***	1	2	3	4	7	10	14
A	562	-	24.4 ^a,b^	25.4	22.82	28.28	25.93	-	-	-	-	-	-	36.1	-	-
	563	-	31.07	28.1	24.75	33.64	22.94	-	-	-	-	-	-	-	-	-
	235	-	34.77	31.86	25.77	35.15	29.01	-	-	-	-	38.62	39.07	36.65	-	-
B	567	-	32.88	28.61	24.38	34.55	25.45	-	-	-	-	-	-	22.84	34.61	-
	230	† ^c^	†	†	†	†	†	†	†	†	†	†	†	†	†	†
	231	-	34.17	27.76	25.82	34.55	23.62	-	-	-	-	-	34.98	34.78	-	-
C	232	-	36.13	28.9	26.59	28.36	27.2	-	-	-	-	-	-	25.92	-	-
	233	-	34.3	29.5	23.33	29.52	23.29	-	-	-	-	-	29.26	35.01	-	-
	234	-	37.13	32.03	27.72	29.38	28.07	-	-	-	-	-	-	26.73	-	-
*Unvaccinated* *Controls*	959	† ^d^	†	†	†	†	†	†	†	†	†	†	†	†	†	†
	564	-	29.01	21.9	21.24	26.44	24.75	-	-	-	38.79	33.44	38.35	22.13	25.83	-
	236	-	26.73	20.56	21.98	26.06	30.28	-	-	-	-	-	31.68	30.49	-	-

* See Table 1 for vaccine identification; ** PCD 0 corresponds to PVD 35; *** PDTD 0 corresponds to PCD 28; -, negative result; ^a^ cycle threshold (Ct) value positive results were considered positive when the Ct value was equal to or less than 45; ^b^ *OIE Manual of Diagnostic Tests and Vaccines for Terrestrial Animals*; † dead; † ^c^ dead post-vaccination day 3; † ^d^ dead post-vaccination day 30.

**Table 4:** Results of BoHV-1 detection using gD and gE real-time PCR of calves immunized with different BoHV-1 marker vaccines, challenge infected with virulent strain of BoHV-1, and treated with DMS.

Group *	Identification No. of Calves	Real-Time PCR Assay **	
		gD	gE
A	562	39.65 ^a,b^	37.44
	563	-	-
	235	39.1	37.3
B	567	23.86	22.27
	230	† ^c^	† ^c^
	231	39.47	33.92
C	232	27.50	26.18
	233	30.65	29.67
	234	23.58	26.80
Unvaccinated Controls	959	† ^d^	† ^d^
	564	22.88	21.81
	236	31.71	29.44

* See Table 1 for vaccine identification; ** Post-DMS treatment day (PDTD) 7; ^a^ Cycle threshold (Ct) value positive results were considered when the Ct value was equal to or less than 40; ^b^ Wernike et al. (2011); †, dead; † ^c^ dead PVD 3; † ^d^ dead PVD 30.

## Data Availability

Not applicable.
